# Draft Genome Sequence of *Methylocaldum* sp. Strain 14B, an Obligate Hydrogen Sulfide-Tolerant Methanotrophic Strain That Can Convert Biogas to Methanol

**DOI:** 10.1128/genomeA.00153-17

**Published:** 2017-04-20

**Authors:** Xiangdong Wei, Xumen Ge, Yebo Li, Zhongtang Yu

**Affiliations:** a Department of Animal Sciences, Agricultural and Biological Engineering, The Ohio State University, Columbus, Ohio, USA; b Department of Food, Agricultural and Biological Engineering, The Ohio State University, Columbus, Ohio, USA; c Department of Environmental Sciences, College of Resource & Environment, Hunan Agricultural University, Hunan, China

## Abstract

The draft genome sequence of *Methylocaldum* sp. 14B, an obligate methanotrophic strain isolated from solid-state anaerobic digestion systems, is reported here. Strain 14B possesses genes for methane oxidation and exhibited tolerance to H_2_S.

## GENOME ANNOUNCEMENT

Biogas is a renewable energy source, but its direct utilization has several limitations due to corrosion caused by H_2_S present in biogas and difficulty with its storage and transportation. Therefore, alternative technologies to utilize biogas are desirable. We recently isolated one strain (*Methylocaldum* sp. 14B) of methanotrophs from a solid-state anaerobic digester fed corn stover (1). 14B is the first methanotroph isolated from anaerobic digesters, and it is an obligate methanotroph closely related to *Methylocaldum szegediense*. 14B can directly convert biogas containing H_2_S to methanol (1), and thus, it can be used in converting biogas into methanol as a liquid fuel or chemical. Species of *Methylocaldum* constitute a novel group of type I methanotrophs, but only the genome of one strain, *Methylocaldum szegediense* O-12 (2), has been sequenced recently (accession no. NZ_ATXX00000000). Here, we present the draft genome sequence of *Methylocaldum* sp. 14B.

*Methylocaldum* sp. 14B was cultured in a mineral medium (3) with methane as the sole substrate. One paired-end library was prepared from genomic DNA using the NEBNext Ultra DNA library prep kit for Illumina (NEB) and sequenced (2 × 300 bp) using MiSeq. Sequence reads were *de novo* assembled using Newbler (4), resulting in 85 contigs (*N*_50_, 133,347 bp; largest contig, 294,260 bp) with an average coverage of 90×. The draft genome of *Methylocaldum* sp. 14B is 4,820,475 bp, with a G+C content of 58.24%. Genes were predicted using Glimmer 3.02 (5–7), and only open reading frames (ORFs) longer than 100 amino acid residues were considered genes. Genes were annotated using the NCBI NR, KEGG (8–11), COG (11, 12), Swiss-Prot (13), GO (14), PHI (15), ARDB (16), VFDB (17), and CAZy (18) databases, and the annotation results were combined to improve gene annotations. Tandem Repeats Finder 4.04 (19), tRNAscan-SE 1.23 (20), RNAmmer 1.2 (21), and Rfam 10.1 (22) were used to identify tandem repeats, tRNAs, rRNAs, and small RNA (sRNA) sequences, respectively. Minisatellite DNA and microsatellite DNA were predicted based on the number and length of the repeat units.

The draft genome contained four rRNA genes (two 5S, one 16S, and one 23S rRNA), 45 tRNA genes, one sRNA gene, 98 minisatellite DNA, 12 microsatellite DNA, 152 tandem repeat sequences, 4,586 genes with predicted functions, and 919 genes coding for hypothetical proteins. MegaBLAST searches (23) of the 14B concatenated genome against the NCBI reference genome database (http://www.ncbi.nlm.nih.gov/genome) revealed that the most closely related genome was that of *Methylocaldum szegediense* O-12 (accession no. NZ_ATXX01000000), with 87% sequence coverage and 95% sequence identity.

Key genes associated with the pathways of the tricarboxylic acid cycle, methane oxidation, sulfur metabolism and relay system, glycolysis and gluconeogenesis, pentose phosphate, oxidative phosphorylation, cell motility, one-carbon assimilation (the serine cycle and the ribulose-1,5-bisphosphate [RuBP] pathways) were identiﬁed, in agreement with the characterization data of this strain (1). Genes encoding enzymes of the methane monooxygenase (particulate), sulfide dehydrogenase, sulfite oxidase and reductase, and sulfur transferase were also detected.

### Accession number(s).

This whole-genome shotgun project has been deposited at DDBJ/EMBL/GenBank under the accession number MSCV00000000. The version described in this paper is the ﬁrst version, MSCV01000000.1.
